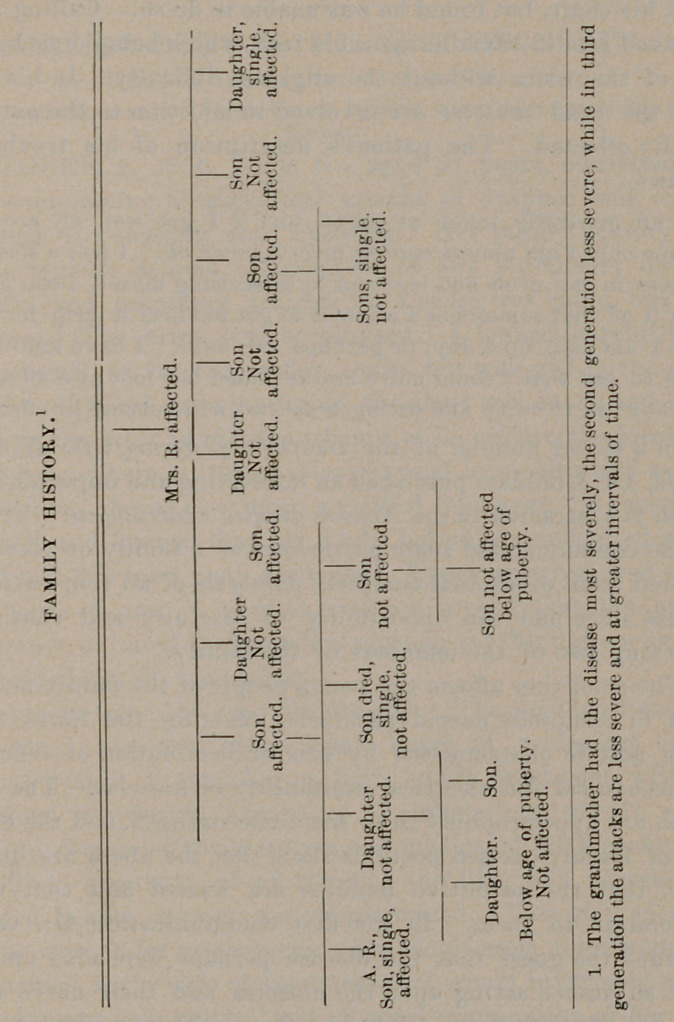# Thomsen’s Disease—A Family History

**Published:** 1897-08

**Authors:** J. C. Clemesha

**Affiliations:** M. D., L. R. C. P., Lond., M. R. C. S., England


### Clinical Reports.

### THOMSEN'S DISEASE—A FAMILY HISTORY.

BY J. C. CLEMESHA, M. D., L. R. C. P., Lond., M. R. C. S., England.

IN JANUARY, 1896, I was called upon to attend A. R., aged 33, suffering from an attack of hemoptysis. I found evidence of consolidation in the right apex, and the like, and the disease has been running its varying course ever since.

On going into his past history he mentioned being subject to the "family complaint" and on inquiry I learned the following: At different intervals during the last twenty years he had been subject to attacks of paroxysmal paralyses, at which times he would be unable to lift his arms or legs ; in severe attacks his head could not be moved. He became afflicted at or about the age of thirteen (puberty) and tells me that all the members of his family, so troubled, were between the ages of thirteen and eighteen when the disease first showed itself.

Dampness and cold increases the liability to attacks, which, by experience, he could tell the onset of by a peculiar clumsiness or uselessness in the hands and feet. Often, by two or three hours' brisk walk, he could abort the oncoming paralysis and frequently in the middle of the night would he arise and go for a long tramp. If, during these walks, "to walk off an attack," he were to stumble and fall down he would be unable to rise without assistance, but when on his feet could go long distances without fatigue.

When the patient was younger these attacks used to come on every three or four weeks. Of late years three months or more will elapse and then the attack will not be nearly so severe or so long in duration as in his younger days. If, an attack coming on during sleep, he happened to have his arms and legs in a state of flexion, on awakening he would be unable to extend his extremities and would have to call some one to put his legs straight.

In most of his attacks his lower extremities were alone affected and he tells how during some years' stay in Winnipeg he had one day walked off an oncoming attack, as he thought, and then went into a hotel to dine. After having dinner he attempted to arise from his chair, but found he was unable to do so. Calling a waiter he asked him to lift him upon his feet, which being done he walked out of the room without the slightest difficulty. In his father's case the head muscles are involved in addition to the extremities usually affected. The patient's description of his trouble is as follows:

> I am generally taken at night, but if I get wet, sit around and become cold I am almost sure to have it come on. I feel a sort of uselessness in my arms and legs and by exercising myself I can generally work it off, but sometimes I allow it to get too firm a grip on me and then I am laid up a day, or perhaps two days. I have known myself to be so bad that I could move neither hand nor foot at 5.30 A. M., yet at 7.30 A. M. to be up and eating breakfast without any ill-effects.

In a recent number of the *Deutsche Zeitschrift für Nervenheilkunde*, Dr. Goldflam published an interesting and important article, which is abstracted in the *Neurologisches Centralblatt.* Five years ago he communicated some particulars of a family of eleven, who suffered from occasional complete paralysis of all four extremities. He has since had the opportunity of carefully and continuously observing two of the members of the family.

The condition affects the young people of the family and shows itself in complete flaccid paralysis, affecting the limbs and the trunk, and is characterised by loss or diminution of reflexes and of mechanical and electrical excitability of muscles. The muscles which are hypertrophied show but little strength, and the examination of freshly excised portions show that the fibers are unusually large, that the primitive bundles are wasted and that there is vacuolation in them. In his first communication Dr. Goldflam hazarded the guess that the disease perhaps depended upon some toxic substance acting upon the muscles and their nerve endings so as to influence them and disturb them. This hypothesis receives a certain measure of support in the fact that a ptomaine-like substance is present in the urine of the patients and that a fairly constant condition of leucocytosis is also present.

Possibly the poison exerts its influence on the muscles that have undergone some change in structure which renders them more susceptible to the influence of the substance. No doubt an analogy exists between such cases and those of Thomsen's disease.

I might, in concluding, remark that the above case seems to present characters that exist alike in Thomsen's disease and in Dr. Goldflam's cases.

329 FRANKLIN STREET, BUFFALO.

## Figures and Tables

**Figure f1:**